# Unusual anogenital apocrine tumor resembling mammary-like gland adenoma in male perineum: a case report

**DOI:** 10.1186/1746-1596-5-42

**Published:** 2010-06-25

**Authors:** Kazuhito Hatanaka, Akihide Tanimoto, Yoshihisa Umekita, Takako Yoshioka, Takuro Kanekura

**Affiliations:** 1 Department of Molecular and Cellular Pathology, Kagoshima University Graduate School of Medical and Dental Sciences, 8-35-1 Sakuragaoka, Kagoshima 890-8544, Japan; 2 Department of Dermatology, Kagoshima University Graduate School of Medical and Dental Sciences, Kagoshima, Japan

## Abstract

A rare case of an apocrine tumor in the male perineal region is reported. A dermal cystic lesion developed in the region between the anus and scrotum of a 74-year-old Japanese male. The cystic lesion, measuring 3.5 × 5.0 cm in size, was lined by columnar or flattened epithelium with occasional apocrine features and supported by a basal myoepithelium lining. A mural nodule, measuring 1 × 1.5 cm in size, protruded into the cystic space and consisted of a solid proliferation of tubular glands with prominent apocrine secretion and basal myoepithelial cells. Immunohistochemical examination showed that the luminal cells were partially positive for gross cystic disease fluid protein 15 and human milk fat globulin 1, and the basal myoepithelial cells were positive for alpha-smooth muscle actin and S-100 protein. Estrogen and progesterone hormone receptors were focally and weakly positive for luminal epithelium. Although no mammary-like glands were present in the dermis around the tumor, this unusual apocrine tumor has been suggested to be derived from male anogenital mammary-like glands and mimic a mammary-like gland adenoma in the male perineum.

## Background

Mammary-like glands (MLGs) are present in the skin of the anogenital region of males as well as females. MLGs are distributed in the periclitoral region, interlabial sulci, fourchette, perineal and perianal regions in females and in the ventral side of the penis, perineal and perianal regions in males [1]. Although eccrine glands develop separately from apocrine glands in embryological considerations [2], the MLGs show intermediate morphology between eccrine and apocrine glands that resemble mammary glands and are originally referred to as anogenital sweat glands [3]. Many skin tumors in the anogenital region, including hidroadenoma papilliferum, apocrine cystadenoma, adenosis tumor, and extramammary Paget's diasease, are now thought to arise from the MLGs [1,4].

We describe a rare apocrine tumor in the anogenital region of a male and suggest that it is derived from male anogenital MLGs.

## Case presentation

**Figure 1:**
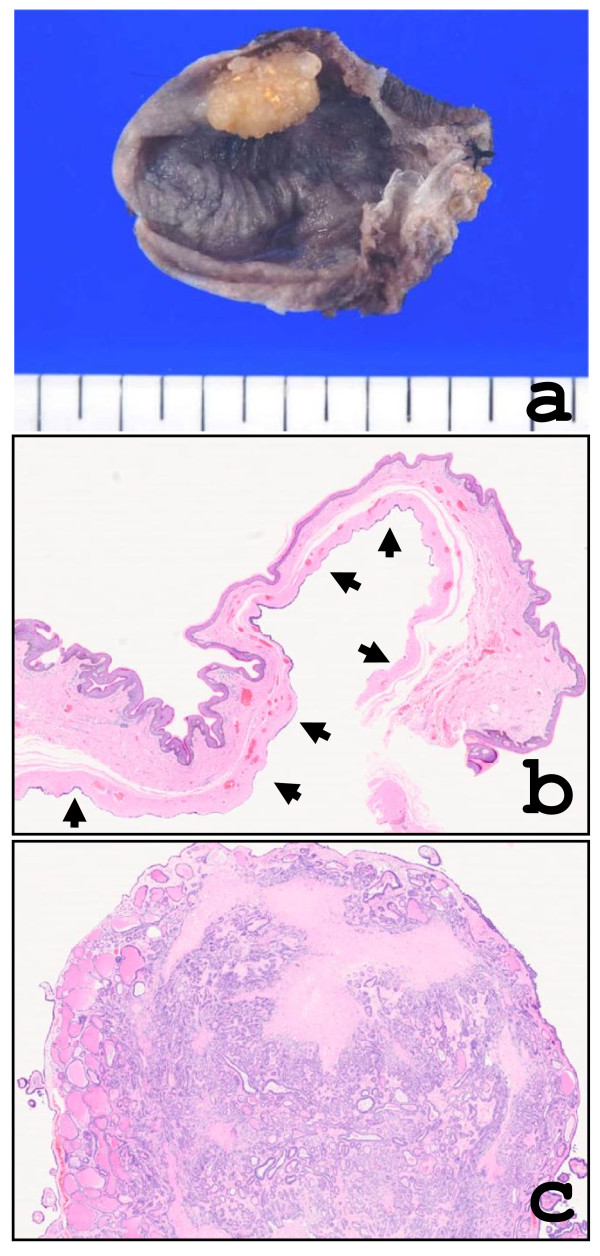
**a) Macroscopic view of the resected specimen showing opened cystic tumor with a polypoid projection into the cystic space**. b) At scanning view, the cystic tumor was located in the dermis (arrow). c) A panoramic view of the intracystic solid portion of the tumor.

A 74-year-old Japanese male presented with a skin tumor between the anus and scrotum. On physical examination, the lesion was 5 cm in maximal diameter and located in the dermis and covered with normal epidermis. The resected lesion, measuring 3.5 × 5.0 cm in size, was a cystic tumor containing fluid content. A yellowish polypoid intra-cystic tumor, measuring 1 × 1.5 cm in size, was projected into the cystic space (Figure 1a).

## Pathological findings

**Figure 2:**
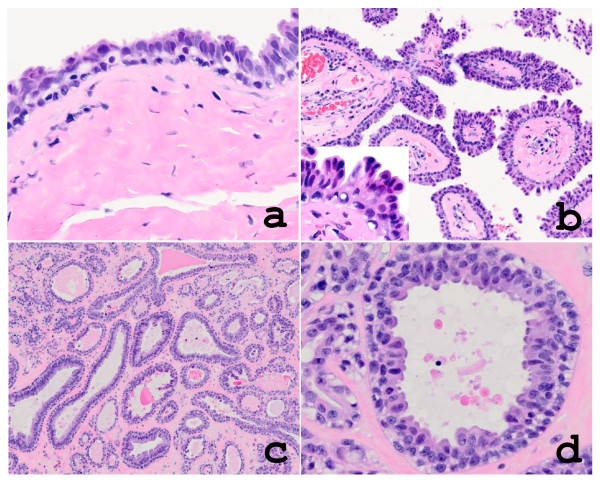
**a) The cyst wall was lined by luminal epithelium with occasional apocrine snouts and basal myoepthelial cells, sitting on the collagenous capsule**. b) Papillary projection of columnar cells with apocrine feature was observed (insert). c) The intracystic polypoid projection consisted of proliferation of glandular structures embedded in collagenous stroma. d) The tubules were lined by luminal columnar cells with apocrine secretion and by basal myoepithelial cells.

**Figure 3:**
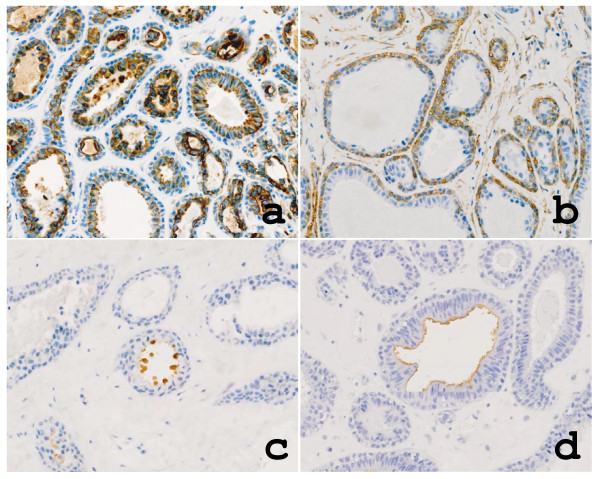
**Immunohistochemical examinations of the tumor cells**. The glandular epithelium was positive for a) cytokeratin (CK7) and the basal myoepithelial cells were positive for b) α-SMA. The luminal surface was partly positive for c) GCDFP-15 and d) HMFG-1.

The epidermis showed no remarkable change. The cystic tumor with no connection to the epidermis was located in the dermis and encapsulated by dense collagenous tissue (Figure 1b). The inner surface of the cyst wall was lined by two rows of cells of luminal columnar to cuboidal or flattened epithelium with occasional apocrine snouts and basal myoepithelial cells (Figure 2a). A low papillary projection of columnar cells with apocrine features was observed in the cystic epithelium (Figure 2b). The intra-cystic polypoid projection (panoramic view in Figure 1c), which was located in the capsular tissue, consisted of a proliferation of glandular structures with hyalinized stroma (Figure 2c). The tubules were lined by tall columnar cells with distinct apocrine snouts and by basal myoepithelial cells (Figure 2d). Immunohistochemistry revealed that the cystic and glandular epithelium was positive for cytokeratin 7 (CK7; Novocastra) and low molecular weight keratin (CAM5.2; Becton Dickinson) (Figure 3a). The myoepithelial cells were positive for alpha-smooth muscle actin (α-SMA; Dako) (Figure 3b) and S-100 protein (Dako). The luminal surface of both cystic and glandular portions was diffusely positive for carcinoembryonic antigen (CEA; Novocastra) and partly for gross cystic disease fluid protein 15 (GCDFP-15; Signet) (Figure 3c) and human milk fat globulin 1 (HMFG-1; Medac) (Figure 3d). Immunoreactivity for estrogen receptor (ER; DAKO) and progesterone receptor (PgR; DAKO) were found focally and weakly in the cystic and glandular epithelium.

Both cystic and glandular parts were lined by two rows of epithelial linings: luminal epithelium with apocrine differentiation and basal myoepithelium. Although normal MLGs were absent in the resected specimen, the diagnosis of an apocrine tumor with apocrine cystadenoma-like and intra-cystic tubular adenoma-like features was made.

## Discussion

Hidroadenoma papilliferum, an adenoma with apocrine differentiation, usually occurs in females but also in males. Another case of vulvar apocrine tumor has been reported to show combined histological features with hidroadenoma papilliferum and syringocystadenoma papilliferum [5]. In the present case, however, no papillary folding or connection to the epidermis, which is a feature of hidroadenoma papilliferum or syringocystadenoma papilliferum respectively [6], was observed but predominantly showed cystic and intra-cystic solid patterns. The cystic part was similar to apocrine cystadenoma (hidrocystoma) and the tubular structure with collagenous stroma in the intra-cystic solid area resembled tubular apocrine adenoma, in which these apocrine tumors usually occur in the face and scalp, respectively [6]. Since the cystic and solid growth of the present tumor was quite unique and did not fit the known histological classification of apocrine tumors, the tumor was initially diagnosed as an unclassified benign apocrine tumor.

The hidroadenoma papilliferum occurs at the anatomical area where MLGs are distributed and is recently recognized as the most common clinical manifestation of MLGs-related tumors in vulvar MLG adenomas [4]. The MLG adenomas, however, demonstrate a marked diversity in histological appearance including tubular, papillary, cystic and solid patterns, and a combination of two or more patterns [4,5,7,8]. Furthermore, as the present case partly exhibits apocrine differentiation, unusual anogenital glandular lesions showing a minor or no apocrine differentiation have been reported under various diagnoses including apocrine cystadenoma, fibroadenoma, ductal adenoma and adenosis tumor in addition to hidroadenoma papilliferum and syringocystadenoma papilliferum [1,4]. Since normal MLGs show intermediate morphology between eccrine and apocrine glands similar to mammary glands [1,3], the recognition of a clinicopathological spectrum of MLGs-related tumors would be very practical in order to describe these tumors showing such histological diversity [4]. In this context, although the MLGs-related tumors are very rare in male [1], the present tumor showing combined cystic and intra-cystic tubular growth with apocrine differentiation (apocrine cystadenoma-like and tubular apocrine-like features) would fulfill the clinicopathologic criteria of MLG adenomas. Recently, the MLGs-related tumors are categorized in the tumors of modified apocrine glands, which further include Moll's gland adenocarcinoma of the eye, ceruminous adenoma or adenocarcinoma of the external auditory canal, and erosive adenomatosis of the nipple [6].

Immunohistochemically, the luminal cells were positive for low molecular weight cytokeratins (CK7 and CAM5.2) and CEA. GCDFP-15 and HMFG-1, markers for apocrine differentiation [9-12], were partially expressed in the luminal cells. On the other hand, the basal cells were positive for α-SMA and S-100 protein, indicating myoepithelial nature. This immunoprofile was very consistent with that in normal MLGs and hidroadenoma papilliferum [1,5], except for focal and weak expression of ER and PgR, which commonly are found in normal mammary glands. Moreover, immunoreactivity for ER was identified in hidroadenoma papilliferum [13]. In males, median raphe cysts, which occasionally show apocrine metaplasia on the luminal surface [14], share anatomical location and histological findings with apocrine cystadenoma [6]. In the present case, however, the cystic wall was supported by α-SMA/S-100 protein-positive basal myoepithelial cells, which differentiates median raphe cyst from a true apocrine tumor. Furthermore, as in the presented case, HMFG-1 expression has been reported to be a useful marker for apocrine tumors but not for median raphe cysts [15].

## Conclusion

We have described a rare apocrine tumor in the male anogenital region. Although normal MLGs were absent in the adjacent tissue, the tumor in the present case demonstrated the same histological and immunohistochemical pattern as MLG adenomas.

## Consent

Written informed consent was obtained from the patient for publication of this case report and any accompanying images. A copy of the written consent is available for review by the Editor-in-Chief of this journal.

## Competing interests

The authors declare that they have no competing interests.

## Authors' contributions

MR, KH and AT participated in conception of the idea and writing of the manuscript. AT, YU, TY and TK performed the histopathological interpretation of the tumor tissue.

All authors have read and approved the final manuscript.

## References

[B1] van der Putte SC Anogenital "sweat" glands. Histology and pathology of a gland that may mimic mammary glands Am J Dermatopathol 1991 13 557 567 10.1097/00000372-199113060-00006 1666822

[B2] Groscurth P Kreyden OP, Boni R, Burg G Anatomy of sweat glands Hyperhidrosis and Botulinum Toxin in Dermatology. Curr Probl Dermatol 2002 Basel: Kager 1 9 full_text

[B3] Woodworth H Jr Dockerty MB Wilson RB Pratt JH Papillary hidradenoma of the vulva: a clinicopathologic study of 69 cases Am J Obstet Gynecol 1971 110 501 508 4325819 10.1016/0002-9378(71)90691-0

[B4] Scurry J van der Putte SC Pyman J Chetty N Szabo R Mammary-like gland adenoma of the vulva: review of 46 cases Pathology 2009 41 372 378 10.1080/00313020902884493 19404851

[B5] Nishie W Sawamura D Mayuzumi M Takahashi S Shimizu H Hidradenoma papilliferum with mixed histopathology features of syringocystadenoma papilliferum and anogenital mammary-like glands J Cutan Pathol 2004 31 561 564 10.1111/j.0303-6987.2004.00176.x 15268713

[B6] Weedon D Weedon D Apocrine tumors Tumors of Weedon's Skin Pathology 2010 3 New Yolk: Churchill-Livingstone Elsevier 779 794

[B7] Obaidat NA Awamleh AA Ghazarian DM Adenocarcinoma in situ arising in a tubulopapillary apocrine hidradenoma of the peri-anal region Eur J Dermatol 2006 16 576 578 17101482

[B8] Kazakov DV Bisceglia M Sima R Michal M Adenosis tumor of anogenital mammary-like glands: a case report and demonstration of clonality by HUMARA assay J Cutan Pathol 2006 33 43 46 10.1111/j.0303-6987.2006.00391.x 16441411

[B9] Mazoujian G Margolis R Immunohistochemistry of gross cystic disease fluid protein (GCDFP-15) in 65 benign sweat gland tumors of the skin Am J Dermatopathol 1988 10 28 35 10.1097/00000372-198802000-00004 2459984

[B10] Tubura A Senzaki H Sasaki M Hilgers J Morii S Immunohistochemical demonstration of breast-derived and/or carcinoma-associated glycoproteins in normal skin appendages and their tumors J Cutan Pathol 1992 19 73 79 10.1111/j.1600-0560.1992.tb01562.x 1556271

[B11] De Viraph PA Szeimies RM Eckert F Apocrine cystadenoma, apocrine hidrocystoma, and eccrine hidrocystoma: three distinct tumors defined by expression of keratins and human milk fat globulin 1 J Cutan Pathol 1997 24 249 255 10.1111/j.1600-0560.1997.tb01590.x 9138118

[B12] Ohnishi T Watanabe S Immunohistochemical analysis of cytokeratin expression in apocrine cystadenoma or hidrocystoma J Cutan Pathol 1999 26 295 300 10.1111/j.1600-0560.1999.tb01847.x 10472758

[B13] Swanson PE Mazoujian G Millis SE Campbell RJ Wick MR Immunoreactivity for estrogen receptor protein in sweat gland tumors Am J Surg Pathol 1991 15 835 841 10.1097/00000478-199109000-00003 1951842

[B14] Otsuka T Ueda Y Terauchi M Kinoshita Y Median raphe (parameatal) cysts of the penis J Urol 1998 159 1918 1920 10.1016/S0022-5347(01)63196-3 9598487

[B15] Ohnishi T Watanabe S Immunohistochemical analysis of human milk fat globulin 1 and cytokeratins expression in median raphe cyst of the penis Clin Exp Dermatol 2001 26 88 92 10.1046/j.1365-2230.2001.00768.x 11260187

